# Bioaerosols at plants processing materials of plant origin—a review

**DOI:** 10.1007/s11356-020-09121-4

**Published:** 2020-05-15

**Authors:** Karol Bulski

**Affiliations:** Department of Microbiology and Biomonitoring, University of Agriculture in Krakow, Krakow, Poland

**Keywords:** Bioaerosols, Bacteria, Fungi, Processing plants, Materials of plant origin

## Abstract

Due to the dynamic development of industry, related to the processing of plant materials and a subsequent significant increase in the number of employees working in this kind of industry, the indoor air quality is of great importance for the human health. The premises of plants processing plant materials are a specific environment, related to exposure to biological agents. The major sources of microbial contamination of premises are employees’ activities and the operation of devices used in the production process, quality of plant materials, technological processes, construction materials, ventilation (air-conditioning) systems, and outdoor air. Biological agents (primarily bacteria and fungi) transported in the air can cause numerous adverse health outcomes in exposed workers.

## Introduction

The increase in interest in plant materials’ products results in a steady increase in employment in the industry, related to the processing of plant materials. This is due to the fact that plant raw materials are increasingly used in many industries, from food processing to cosmetology or medicine. In recent years, attention has increasingly been drawn to the fact that employees of plants processing materials of plant origin may be exposed to many harmful factors. These factors are chemical and biological pollution, mechanical vibrations, noise, electromagnetic fields, lighting, static electricity, and variable microclimate. Due to the fact that the production rooms are a specific environment, the concerns expressed by employees are related to exposure to biological aerosols. This is due to the fact that we still do not know enough about the relationship between the exposure to bioaerosols and adverse health effects. There is a lack of global generally accepted limit values of concentration of bioaerosols and methodological recommendations, related to the control of these kinds of threats. In the indoor air of plants processing plant materials, any microbiological agent present in the air is undesirable and can be considered as a contaminant. In this type of plant, it is primarily bacteria, microscopic fungi, viruses, plant pollen, seeds, and products produced by microorganisms, e.g., endotoxins, glucans, fungal metabolites and allergens, enzymes. These particles (suspended in the air) are present usually in low concentrations, so they do not pose a threat to the humans (their presence can be considered as natural). The problem of the contamination may appear when the level of microbial contaminants in the air rises above a certain limit, which is considered as a natural background for the environment.

Microbiological contamination of indoor air may result from microbial emissions in the room or may come from the external environment. Bioaerosols’ levels usually increase when rooms are occupied and humans have been reported to be a source of microorganisms in settled dust samples (Adams et al. 2015; Brągoszewska 2019). In industry, an additional source of biological aerosol can be production and processing (Kim et al. 2018). Unfortunately, individual exposure levels and susceptibilities are highly variable. That is why an inhalation dose has not yet been specified. It is difficult to characterize the actual risk to workers from exposure to biological aerosols resulting from their work activities (Brągoszewska and Biedroń 2018). Nowadays, more research is needed to precisely describe the role of biological aerosols in the initiation or development of diverse symptoms and human diseases (Jiayu et al. 2019).

The author decided to prepare a review paper, which is a kind of summary and describing the environmental problem of bioaerosol in plants processing raw materials of plant origin, including possible sources of biological aerosols in this type of workplace and their potential impact on the worker’s health.

## Bioaerosol—definition and characteristics

As defined, the term “bioaerosol” or “biological aerosol” means a collection of biological particles dispersed in air or another gas phase. Bioaerosol particles can be (Górny 2010; Brągoszewska et al. 2018; Ławer 2019) as follows:

- Single spores of microorganisms, pollen, bacterial vegetative cells, and viruses;
- Aggregates composed of several spores of microorganisms or bacterial vegetative cells;
- Aggregates formed by several spores of microorganisms or bacterial vegetative cells with other biological material (e.g., mammalian allergens);
- Products of microbial origin, fragments of bacterial cells, or fungal spores (e.g., endotoxins, mycotoxins);
- Multigrain structures composed of non-biological particles transporting material of biological origin (e.g., endotoxin particles connected with granular aerosol particles).

The composition, size, or population of microorganisms forming bioaerosol depends on the source, dispersion mechanisms in the air, and environmental factors, such as the type of organism, viability, growth chase, and resistance to electromagnetic radiation (Niemiec and Zamorska 2002; Kaiser and Wolski 2007; Nourian et al. 2007).

Biological aerosols are omnipresent in the natural environment. Biological particles can be released into the air from almost any natural or artificial surface. Bacteria and fungi can get into the atmosphere as a result of their removal from plant and soil surfaces, due to the wind or thermal convection processes, and after spontaneous or rain forced emissions from natural water reservoirs. The human activity also has a big impact on the qualitative and quantitative composition of the biological aerosol. The quantitative and qualitative diversity of microorganisms in the air and their distribution in different regions of the world may result from differences in the density and size of the human population and industrial activity (Redford and Fierer 2009; Heo et al. 2010; Bowers et al. 2012; Polymenakou 2012).

The range of aerodynamic diameters of bioaerosol particle sizes from nanometric (e.g., bacterial endotoxins, 30–50 nm), through submicron (e.g., fragments of bacterial or fungal cells), to particles whose diameter can reach from several dozen to over 100 μm. The biological fraction of bioaerosols in the atmosphere consists of pollen (17–58 μm), fungal spores (1–30 μm), bacteria (0.25–8 μm), viruses (< 0.3 μm), and plant and animal fragments, representing 10 to 30% of the volume of all suspensions in this environment. Bacteria in the air are most often attached to organic or inorganic particles ranging in size from ≤ 0.65 to ≥ 7.0 μm, with an average particle size ≥ 2.1 μm in diameter. On the other hand, fungi usually have a size below 10 μm (Grajewski and Twarużek 2004; Błaszczyk 2010; Estillore et al. 2016; Mazurkiewicz-Zapałowicz et al. 2016).

Microorganisms that are part of bioaerosols cannot grow during airborne transport—they are only able to survive in the air for some time. It depend on their properties and environmental conditions. This is influenced by the nature of the environment (access to nutrients, physical, and chemical factors of environmental stress) and the individual characteristics of microorganisms, including the particle size of bioaerosols (Pillai and Ricke 2002; Kaiser and Wolski 2007; Nourian et al. 2007; Menetrez et al. 2010; Puspita et al. 2012). The small components of bioaerosol retain their viability in the environment longer than larger microorganisms. Live persistent forms (e.g., bacterial endospores) can survive in the air for a long time, and the most sensitive, vegetative forms die quickly (Table 1). Due to the relatively small size of the cell, the most adapted forms of microorganisms are cocci and bacteria whose cells are covered with a layer of mucus or produce yellow and red carotenoid dyes that protect them from harmful UV radiation (Gotkowska-Płachta et al. 2008; Gatchalian et al. 2010; Barabasz et al. 2011; Galperin and Yutin 2013).

**Table 1:** The survival time of selected bioaerosol components in the air (Górny 2004)

Microorganism	Survival time in the air
*Aspergillus, Penicillium*	Over 12 years
Mite allergens	Decay within a few months
Flu viruses	Up to 3 weeks
*Escherichia coli*	Dies within 30–60 min
*Streptococcus faecalis*	Dies within 30–60 min
*Legionella pneumophila*	Up to 15 min

## The impact of bioaerosol on the human health

Under normal environmental conditions, at low bioaerosol concentrations, most microorganisms do not pose a health risk. Unfortunately, some of them may be pathogenic, allergic, or toxic. Microbiological aerosols are of the greatest epidemiological importance (Górny 2019). Their presence in the environment can lead to adverse health effects in people, from simple irritation, through allergic reactions, to the occurrence of infections or toxic reactions. The health effect caused by inhalation of various types of biological particles depends mainly on their size, chemical composition, microbiological properties, and the place of their deposition in the respiratory system. It is associated with many diseases of social significance, especially respiratory diseases (Flannigan and Miller 1994; Douwes et al. 2003; Sykes et al. 2007; Roy and Reed 2012; Walser et al. 2015; Górny 2019). The threat from biological factors is associated with the performance of a specific profession and the presence or properties of each factor (Dutkiewicz and Górny 2002; Dutkiewicz 2004; Nguyen et al. 2017). Diseases whose etiological factors are transmitted by the air include the following (Chmiel et al. 2015; Ebisz et al. 2016; Prażak and Kowalska 2017):

- Viral: chickenpox, influenza, mononucleosis, rubella, mumps (parotitis), and shingles, meningitis;
- Bacterial: bronchitis and pneumonia, rhinitis and bronchitis; pulmonary tuberculosis, diphtheria, whooping cough, and sclerosis;
- Fungal: lung aspergillosis, lung mucormicosis, lung cryptococcosis, bronchial mycosis, lung geotrychosis, fungal pneumonia, pleural mycosis, and others.

It is important that the dispersion of the plant, animal, and human pathogens and allergens has serious implications for agriculture and public health (Fröhlich-Nowoisky et al. 2016). Threats resulting from the presence of microorganisms in the air are not only limited to impact on the health of exposed persons (viral, bacterial, fungal, allergic diseases, poisoning with endotoxins and mycotoxins), but also to microbial contamination of products produced in the industry (food, pharmaceutical, cosmetics) and agriculture (crop production, animal production) (Chmiel et al. 2015). The threat is caused not only by the presence of pathogenic microorganisms or toxins in the air, but also by an excessive number of saprophytic microorganisms, especially if their composition is not diversified (Cabral 2010; Gładysz et al. 2010). Harmful microbiological agents can enter the human body by inhalation, direct contact (through the damaged skin or mucous membranes), or by ingestion. Erogenous route and the inhalation of bioaerosol with a high concentration of microorganisms, toxins, or allergens are of paramount importance in the spread of harmful biological agents in the work environment. The digestive tract is not typical for occupational infections (Dutkiewicz and Jabłoński 1989; Mazurkiewicz-Zapałowicz et al. 2016).

The point of the theoretical maximum penetration depth, i.e., how deep in the respiratory system the particle will reach, depends on the aerodynamic diameter of microorganisms. Bioaerosol particles with an aerodynamic diameter (Górny 2019):

- Below 0.65 μm reach the alveoli region;
- 0.65–1.1 μm reach the pulmonary bronchioles area;
- 1.1–2.1 μm reach the end bronchioles area;
- 2.1–3.3 μm reach the secondary bronchial area;
- 3.3–4.7 μm reach the region of the trachea and primary bronchi;
- 4.7–7.0 μm reach the throat region;
- 7.0–11.0 μm reach the nasal passages;
- Above 11.0 μm practically do not penetrate deep into the respiratory system.

Also, allergies can be one of the diseases caused by biological factors. About 100 species of fungi are associated with the symptoms of allergic respiratory diseases. These are usually species such as *Penicillium notatum*, *Aspergillus fumigatus*, or *Cladosporium herbarum* (Fischer and Dott 2003; Helbling and Reimers 2003; Mazurkiewicz-Zapałowicz et al. 2016; Lang-Yona et al. 2016). At low concentrations in bioaerosol, most of the bacteria do not pose a threat to human health, but some of them may exhibit allergenic properties. Bacterial endotoxins and (1-3)-β-d-glucans of fungal origin, found in bioaerosol, may cause upper respiratory tract inflammation, flu-like symptoms, or bronchial asthma (Krajewski et al. 2000; Nielsen et al. 2000; Niemiec and Zamorska 2002; Cyprowski 2007; Ławniczek-Wałczyk and Górny 2010; Gołofit-Szymczak and Górny 2017). The previous analysis of the results showed that in residential, school, and office environments, the mean endotoxin loads in settled floor dust varied between 660 and 107.000 EU m^-2^, 2180 and 48.000 EU m^-2^, and 2700 and 12.890 EU m^-2^, respectively (Salonen et al. 2016).

In industry, the workers often do not have knowledge about health hazards in their work, ignoring the wearing of protective clothes, so there could be a risk to their safety and health (Brągoszewska 2019).

## Bioaerosols in the premises of plants processing materials of plant origin

The main source of biological particles in indoor air are living organisms: people, animals, plants, and materials collected in buildings or external air penetrating the rooms (Pasanen and Kasanen 2000; Gołofit-Szymczak et al. 2013; Chmiel et al. 2015; Małecka-Adamowicz et al. 2019). Analyses carried out in various countries indicate that in most cases, the concentration of microbiological contaminants in the indoor air is higher than in the outdoor environment (Brągoszewska et al. 2020; Li et al. 2020). Also, the quantitative and qualitative composition of bioaerosol in various types of interiors is more stable. This is especially important because we spend more and more time indoors. It is estimated that people spend between 80% and 95% of their time in an indoor environment during their lifetime. An adult performs approx. 20–22 thousand breaths per day, breathing in over 10 m^3^ of air with pollutants (Dacarro et al. 2003; Cabral 2010). There are a lot of studies that investigated human-to-human transmission via expiratory droplets in indoor environments with a view to minimizing it, but we have to remeber that there are also other airborne pathways that can transmit microorganisms (Lai et al. 2018).

In the industrial environment, the upmost impact on the bioaerosol concentrations have employees’ activities and the operations of using devices. This may cause air movement and release the biological particles during the production process. An important source of microorganisms in the industrial work environment are technological processes such as wastewater treatment, waste collection and processing, and biomass extraction and processing (Bawiec et al. 2016; Vantarakis et al. 2016; Patil and Kamble 2017; Han et al. 2018; Paśmionka 2020). In urban waste water, apart from typically environmental microorganisms, there may also be pathogenic species from the genera *Streptococcus*, *Helicobacter*, *Legionella*, or *Salmonella*. These microorganisms can be released into the air and pose a serious threat to workers’ health. Gram-negative enterobacteria from the Enterobacteriaceae family, Gram-positive cocci, and staphylococci are extremely important. Bacterial endotoxin, produced by Gram-negative rods from the Enterobacteriaceae family, may pose a significant threat to worker’s health during biomass processing. Also, during large-scale biomass, burning emissions has implications for endotoxin exposure on human health (Rajput et al. 2017; Wei et al. 2019). Mold fungi of the genera *Aspergillus*, *Penicillium*, *Alternaria*, *Scopulariopsis*, and *Trichoderma* as well as mesophilic and thermophilic actinobacteria may be present in organic dust as a result of biomass processing (Kalogerakis et al. 2005; Buczyńska et al. 2007; Ziajka 2008; Chen and Hildemann 2009; Gołofit-Szymczak and Ławniczek-Wałczyk 2011). It should be emphasized that mycological pollution of both indoor and outdoor air is correlated with the concentration of dust in the air of buildings (Grzyb and Lenart-Boroń 2020).

The air quality at the production areas of plants processing plants depends primarily on the type and microbiological purity of processed materials of plant origin. Microbiologically contaminated plant materials (e.g., cereals or herbs) can contribute to the deterioration of air quality in halls and warehouses (places of processing and packaging). In 25% of cases, plant materials are responsible for microbial pollution of the air (Ziajka 2008). Soil has a significant impact on the microbiota of plant materials—habitat for all groups of microorganisms, represented by aerobes and anaerobes, as well as vegetative and spore forms. The largest microbiological contamination of soil is observed in the surface layer. During cultivation, harvesting, storage, and processing of grain employees may be exposed to harmful bioaerosol. Grain ripening in spikes or harvesting and stored in large quantities with high humidity, subjected to mechanical damage (from aggressive harvesting technique and machine threshing), is a very good substrate for the growth of mold fungi. Machine processing of grain can generate very large amounts of organic dust, which is an important risk factor for employees of plant processing plants (Soroka et al. 2008; Juszczak 2011).

Several dozens of bacterial species can occur in the air of the premises of plants processing plant materials. Gram-positive cocci from the *Staphylococcus* and *Streptococcus* genera, and Gram-negative bacteria (*Pantoea*, *Alcaligenes*, *Acinetobacter*), which occur on the surface of many plants (e.g., on cereal grains) can predominate in indoor air. Also, actinobacteria (*Streptomyces*, *Thermoactinomyces*) and mold fungi may be present in the air, especially predominate genera such as *Aspergillus*, *Penicillium*, and *Alternaria* (Dutkiewicz and Mołocznik 1998). The composition of the air microbiota can be different. It depends (like the concentration of microorganisms) on the number of employees, the impact of the external environment, pollution of plant raw materials, and microclimatic conditions. At one plant processing plants, the number and type of microorganisms in the air can be diverse in each production hall and warehouse (Gutarowska and Piotrowska 2007; Bulski et al. 2017). The microbiological quality of the air in this type of interior may change during the production cycle, depending on the activity of employees and the operation of production machines (Jakubczyk 1999). Receiving and unloading plant materials provided by the supplier, drying excessively moist material, weighing, screening cereal grains, preparing a plant mix, or packaging the finished product may have particular relevance. These activities are related to release the organic dust into the air. The most harmful biological factors occurring in organic dusts (e.g., from cereals or hay) include bacteria and fungi, as well as the allergenic and immunotoxic substances (endotoxins, glucans, mycotoxins) (Gwo-Hwa and Chih-Shan 1999; Wołejko et al. 2016).

An important factor that affects microbial air pollution in industrial environment is hygienic and sanitary rooms condition. The presence of organic dust during production, deposition of pollutions in the crevices of the floor or walls, the growth and presence of fungi on building constructions or equipment in production halls, and the development of biofilm on production surfaces, devices, machines, or tools are examples of “hygienic neglect” in processing plants. It contribute to the increase in the number of microorganisms in the air of the internal environment (Gutarowska and Piotrowska 2007). Also, secondary sources, which are extremely difficult to reveal and detect, can significantly affect the maintenance of adequate air hygiene in the production environment. Frequently contaminated and neglected ventilation or air conditioning systems can also be a source of microorganisms spreading in the air of production halls (Kołożyn-Krajewska 2001; Gutarowska and Piotrowska 2007). The exposure to harmful biological agents may change depending on the indoor construction and equipment, ventilation system and air movement, occupant density and activity, and cleaning procedures (Kozdrój et al. 2019). Additionally, aerobiologists and building scientists should cooperate to determine how building characteristics and features (e.g., air-exchange rate, ventilation process) influence indoor biological aerosol concentrations (Prussin et al. 2015).

In plants processing materials of plant origin, fungi constitute a separate problem. This is due to the specifics of production—when the plant materials (e.g., cereals or herbs) are processed, the air inside the halls can be dry or dusty. In these circumstances, xerophilic molds may occur, especially species from the genera *Alternaria*, *Cladosporium*, *Penicillium*, *Aspergillus*, *Rhizopus*, and *Mucor* are the most frequently represented. It may be the most important cause of allergies (Pastuszka et al. 2000; Obtułowicz 2001; Piotrowska et al. 2001; Górny and Dutkiewicz 2002; Herbarth et al. 2003; Stryjakowska-Sekulska et al. 2007; Simon and Duquenne 2014). In conclusion, the workplace safety of plants processing materials of plant origin is dependent on understanding and controlling of the production or packaging process and other operations that can lead to the generation and dispersion of biological aerosols in the air (Mirskaya and Agranovski 2018).

## Harmful biological agents—acceptable concentrations in the work environment

The quantitative interpretation of the results of bioaerosol measurements in the indoor environment is difficult. This is due to the lack of universally acceptable hygiene standards for harmful biological agents. The main reason for this situation is the inability to determine the close correlation between the dose of a harmful biological agent and the health effect. In addition, the immunity of the human body is an individual mark. However, in Poland, the Polish Panel of Experts of Biological Factors presented the recommended values of permissible concentrations of harmful biological agents in the work environment (Table 2). These values were determined by repeated measurements of harmful biological agents concentrations. It allows to assess the degree of pollution of the studied environment and determine what is “typical and acceptable,” and what is “unusual and unacceptable.”

**Table 2:** Proposals for acceptable concentrations of microorganisms in the air according to the Polish Panel of Experts of Biological Factors (Augustyńska and Pośniak 2016)

Microbiological factor	Permissible concentration	
	Working spaces contaminated by organic dust	Living quarters and public buildings
*Mesophilic bacteria*	1.0 × 10^5^ cfu m^-3^*	5.0 × 10^3^ cfu m^-3^
*Gram-negative bacteria*	2.0 × 10^4^ cfu m^-3^*	2.0 × 10^2^ cfu m^-3^
*Thermophilic actinobacteria*	2.0 × 10^4^ cfu m^-3^*	2.0 × 10^2^ cfu m^-3^
*Fungi*	5.0 × 10^4^ cfu m^-3^*	5.0 × 10^3^ cfu m^-3^
*Bacterial endotoxin*	200 ng m^-3^ (2000 EU m^-3^)	5 ng m^-3^ (50 EU m^-3^)

*cfu* colony-forming units, *EU* endotoxic unit

*For respirable fraction, the proposed values should be halved

Until now, microbiological analyses of air showed that the concentrations of bacterial and fungal aerosols in plants processing plant materials usually range from 10^3^ to 10^6^ cfu m^-3^ (Jain 2000; Dutkiewicz et al. 2000, 2001; Dutkiewicz 2005; Góra et al. 2004; Krysińska-Traczyk et al. 2005; Skórska et al. 2005; Tsapko et al. 2015; Aringoli et al. 2012). The activity like processing agricultural products (grain, hay, herbs) can be responsible for worker’s exposure to viable microorganisms or their fragments and metabolites. High levels of exposure to bioaerosols were detected for sawmill workers and during vegetable or fruit processing (Mayer et al. 2016; Bulski et al. 2017; Mirskaya and Agranovski 2018). Reeb-Whitaker and Bonauto (2014) indicate that occupational exposure to hop dust causes respiratory diseases in workers involved in all phases of the hop harvest and processing. Douwes et al. (2003) pay attention to studies describing a potential exposure to asthmatic diseases in workers during potato processing. Composting of organic waste also can generate bioaerosols which have been associated with a range of acute and chronic adverse health effects and diseases (Robertson et al. 2019).

## Summary

In recent years, biological factors are becoming a serious problem of occupational medicine and public health. Employees working in plants processing plant materials should be informed of possible health hazards arising from inhalation of polluted air. Preventive measures should be identified, through appropriate room architecture and technical equipment, because it can reduce workers’ exposure to harmful biological agents. In environment of plants processing plant materials, it is necessary to monitor microclimatic conditions in production rooms. Correctly designed, efficient, and regularly monitored in terms of hygienic quality ventilation is also equally important.
